# Health insurance purchase intentions in the past decade: a systematic review and future research directions

**DOI:** 10.1186/s12913-025-12917-0

**Published:** 2025-06-02

**Authors:** Zhangwei Zheng, Hafizuddin-Syah B.A.M, Hafizah Omar Zaki, Qin Lingda Tan

**Affiliations:** Faculty of Economics and Management, Universiti Kebangsaan Malaysia, Bangi, Selangor 43600 UKM Malaysia

**Keywords:** Health insurance, Consumer purchase intention, Influencing factors, Systematic review, TCM-ADO framework

## Abstract

**Background:**

Health insurance plays a critical role in reducing financial burdens and improving healthcare access. However, misconceptions about risks and costs often lead to poor decision-making and low coverage, even in major economies. Understanding the factors influencing health insurance purchase intentions is essential for expanding coverage and addressing healthcare disparities. This study systematically reviews these factors to identify key patterns and research gaps.

**Methods:**

A systematic review was conducted using the TCM (theory-context-methodology) and ADO (antecedent-decision-outcome) frameworks. Forty-eight studies published from 2014 to 2023 were analyzed to categorize influential factors based on theoretical models, geographic contexts, methodological approaches, and behavioral antecedents, decisions, and outcomes.

**Results:**

A total of 141 influential factors were identified across the reviewed studies, with a steady increase in publications over the decade. The TCM framework revealed the predominant use of the Theory of Planned Behavior, while research focused mainly on China, the United States, and India. The ADO framework highlighted behavioral antecedents as the most significant, followed by individual and financial factors, with purchase intention being the primary decision variable.

**Conclusions:**

This review synthesizes current research on health insurance purchase intentions, identifying significant theoretical fragmentation and geographic disparities. It also provides recommendations for future research to explore underrepresented regions and emerging trends, with implications for expanding health insurance coverage and promoting healthcare equity.

## Introduction

Health insurance is essential for reducing illness-related financial burdens, preventing poverty, [1, 2] and improving access to medical services [3, 4]. It also enhances perceptions of healthcare quality, improving overall health and living standards [5]. Research shows that health insurance reduces mortality rates [6] and mitigates healthcare access disparities driven by economic differences [7, 8]. However, consumers often misjudge risks, leading to poor insurance decisions [9, 10]. Risk assessment is frequently based on subjective perceptions rather than objective probabilities, [11, 12] and unclear healthcare costs further complicate the choice of adequate coverage, [13, 14] ultimately affecting health insurance purchase intentions [15].

Moreover, the health insurance industry plays a critical role in promoting economic growth and enhancing social welfare [16, 17]. By reducing the financial strain on state-sponsored social insurance, it fosters healthcare innovation, improves public health, and boosts national productivity by minimizing illness-related labor losses [18, 19]. However, even in major economies, insurance penetration remains low. For instance, China, the world’s second-largest economy, had a health insurance penetration rate of just 0.72% in 2022 [20].

To address this issue, this study examines the factors influencing consumers’ health insurance purchase intentions through a systematic review of research conducted over the past decade. Understanding these factors is crucial for expanding coverage, reducing healthcare disparities, and providing insights for scholars, policymakers, and practitioners.

Previous studies have explored demographic and socioeconomic factors such as age, gender, and education, [21–23] as well as family-related variables, including dependents, family size, and family behaviors [24–26]. Risk-related factors, such as perceived risk and risk propensity, [27, 28] and expectancy-related factors, including perceived economic instability and future risks, [26, 29] have also been examined. Additionally, product-related factors, such as cost, benefits, coverage, and service quality, have been analyzed [30–33].

However, existing research presents notable limitations. Many prior reviews focused on specific contexts, such as community-based health insurance in low- and middle-income countries, [34–36] or determinants in individual countries like India [37] and Malaysia [38]. Niche topics, such as long-term care insurance, [39] social health insurance, [40] and behavioral economics, [41] have also been explored separately, resulting in fragmented insights that limit a comprehensive understanding of health insurance purchase intentions across broader contexts.

This review addresses the following key research questions: What are the primary factors influencing health insurance purchase intentions across different socioeconomic and geographical contexts? How have theoretical frameworks and research methodologies shaped the understanding of health insurance purchasing behavior over the past decade? By synthesizing recent academic literature, this review provides a comprehensive and comparative perspective on health insurance purchase intentions. This includes various health insurance subtypes and geographic contexts without restrictions, offering a holistic analysis that contrasts with prior studies’ narrow foci. The review also emphasizes the theoretical, contextual, and methodological gaps, such as the fragmented use of behavioral models (e.g., Theory of Planned Behavior) and the limited focus on lower-income countries. Addressing these gaps lays a foundation for future research, advocating for more diverse research designs and deeper exploration of underexamined factors.

In addition to advancing academic understanding, this review offers practical contributions by synthesizing insights that inform policymaking and market strategies. For policymakers, the findings highlight key determinants of consumer insurance decisions that can shape interventions aimed at increasing health insurance coverage, particularly in underserved regions and vulnerable populations. For practitioners, the review underscores the importance of tailored product design, clear communication of benefits, and enhanced service quality to meet consumer needs and address behavioral barriers. By presenting actionable recommendations, this review bridges the gap between research and practice, contributing to more effective strategies for improving health insurance uptake and reducing disparities in access.

The subsequent structure of this paper includes an introduction to the research methodology, followed by the presentation of results and discussion. It then offers suggestions for future research directions and concludes with the final summary.

## Method

This study conducted a framework-based systematic literature review following a previously established classification [42]. It applied a combined ADO (antecedent-decision-outcome) and TCM (theory-context-methodology) framework, integrating key findings from the reviewed articles and providing a systematic structure [43]. This integrated approach addresses the limitations of individual frameworks and offers a more comprehensive overview of health insurance literature [10, 44].

Following the Preferred Reporting Items for Systematic Reviews and Meta-Analyses (PRISMA) guidelines, [45] this review adhered strictly to established procedures. Literature identification began in mid-December 2023, covering studies published between 2014 and 2023. Scopus, Web of Science, and ScienceDirect were chosen to ensure a robust search for high-quality, peer-reviewed literature while minimizing redundancy. Focusing on the past decade ensured relevance, as factors influencing purchase intentions may have shifted over time. The search strategy used core keywords such as ‘health insurance’, ‘purchase’, and ‘intention’, along with their synonyms, and was tailored to each database’s advanced search features (Table 1). A total of 874 articles were initially identified across the three databases. After deduplication using Endnote, 766 articles remained for manual screening.

**Table 1:** Search strategy used in this study

Database	Search strategy	Search results
Scopus	(TITLE-ABS-KEY(purchas* OR buy* OR acquire OR obtain OR shopping OR procure OR decision-making) AND TITLE-ABS-KEY(intent* OR willingness OR desire OR motivation) AND TITLE-ABS-KEY(health insurance OR medical insurance OR health plan* OR medical plan* OR health coverage OR medical coverage OR healthcare coverage OR health scheme* OR medical scheme*)) AND PUBYEAR > 2013 AND PUBYEAR < 2024	610
ScienceDirect	Year: 2014–2023, Title, abstract, keywords: (medical insurance OR health insurance) AND (purchase OR buy OR obtain OR decision-making) AND (intention OR willingness OR motivation)	42
Web of Science (WOS)	((TS=(medical insurance OR health insurance OR health plan* OR medical plan* OR health coverage OR medical coverage OR healthcare coverage OR health scheme* OR medical scheme*)) AND TS=(purchas* OR buy* OR acquire OR obtain OR shopping OR procure OR decision-making)) AND TS=(intent* OR willingness OR desire OR motivation) and 2023 or 2022 or 2021 or 2020 or 2019 or 2018 or 2017 or 2016 or 2015 or 2014 (Publication Years)	222
Total	-	874

Four authors collaborated to screen the literature, reducing individual bias and establishing unified selection, inclusion, and exclusion criteria (Table 2). Disagreements were resolved through group discussions. The title and abstract review resulted in the exclusion of 698 articles that did not meet the criteria. The high exclusion rate was expected, as the search covered “title, abstract, and keywords” to ensure a comprehensive collection of relevant literature. Many excluded articles primarily focused on medical topics, such as drug cost-effectiveness, disease-related financial burdens, treatment acceptance, and vaccination willingness. Additionally, some non-medical articles were excluded for discussing unrelated topics, such as macro-level insurance industry issues or non-health-related insurance purchases. Ultimately, 68 articles were selected for full-text review.

**Table 2:** Inclusion and exclusion criteria for study selection

Inclusion criteria	Exclusion criteria
1. Articles discussing the influencing factors of health insurance purchase intentions.	1. Articles for which the original full text cannot be found.
2. Articles using a non-medical and consumer perspective	2. Articles not focus on the influencing factors of health insurance purchase intentions.
3. Articles using microdata.	3. Articles using non-consumer perspective or utilizing macrodata only.
4. Empirical articles.	4. Articles that are non-empirical articles (e.g. book chapters, reviews, conference abstracts).

During the full-text review phase, three articles were excluded due to unavailability of full text. Fourteen were omitted for focusing on health insurance attributes and preference reasons rather than purchase intention. Additionally, one article was excluded for being outside the business studies domain, one was a conference paper, and another a literature review, making them unsuitable for inclusion. Consequently, 48 articles were selected for this systematic review.

**Fig. 1:**
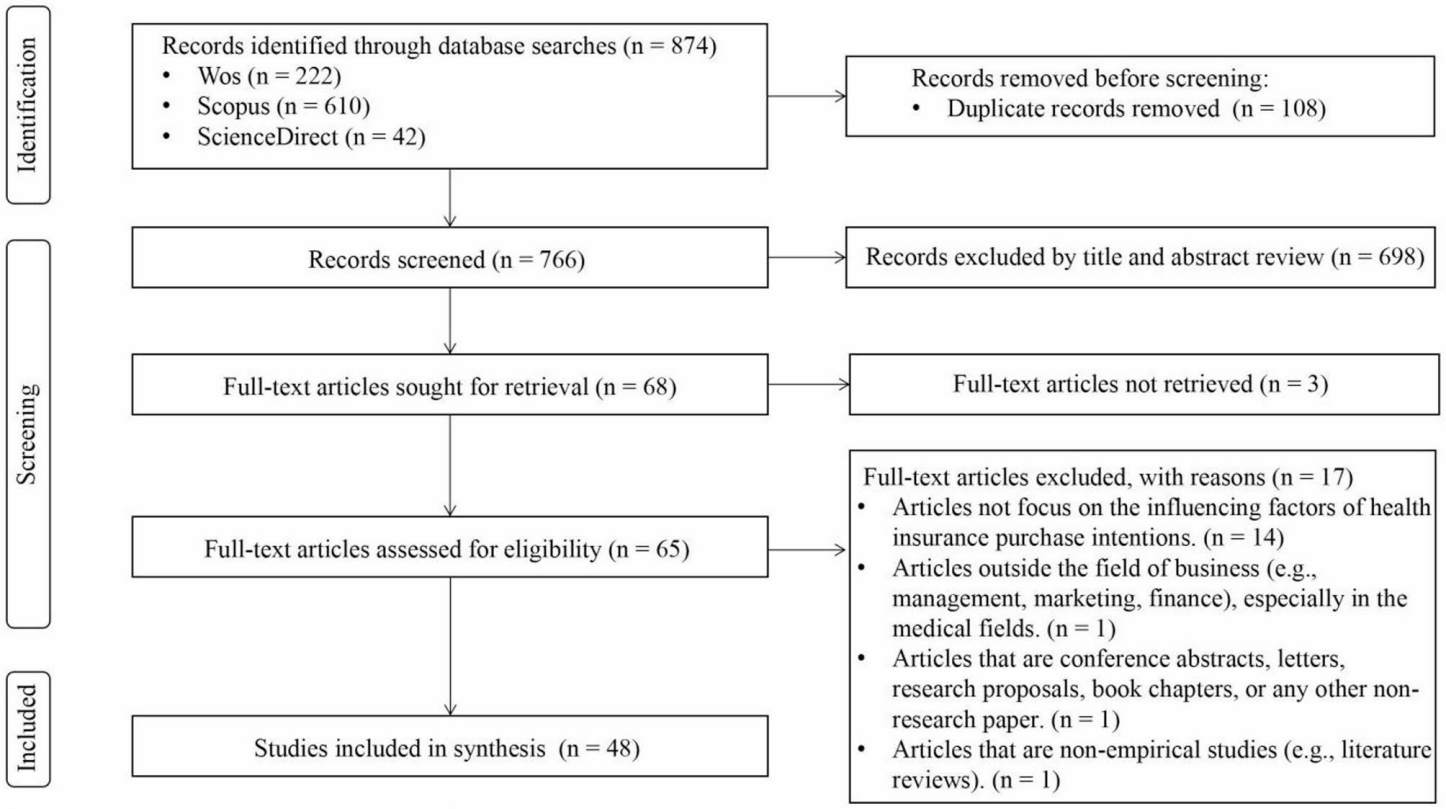
PRISMA flow diagram

In the data extraction and synthesis stage, this study utilized the combined TCM and ADO frameworks to extract key information, including title, publication year, journal, theory, country, data collection and analysis methods, sampling approaches, influencing factors, decisions, outcomes, and health insurance types from 48 selected journal articles published between 2014 and 2023. This approach provides a structured foundation for the content analysis presented in the following sections. The detailed workflow of the literature search process is illustrated in Fig. 1.

## Publishing trends

**Fig. 2:**
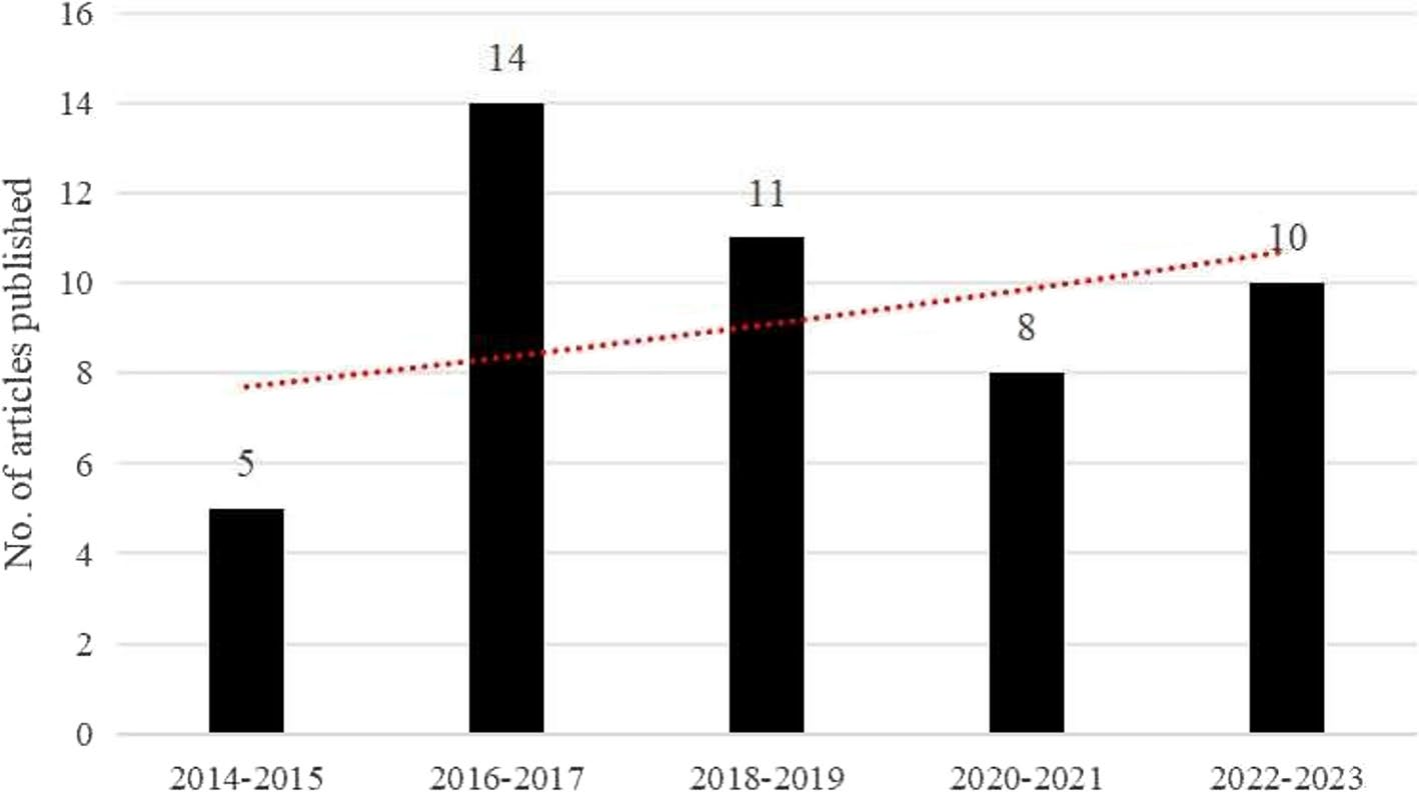
Publishing trends

Figure 2 illustrates the publishing trends of studies reviewed from 2014 to 2023. The red trendline shows that, despite some fluctuations, the overall number of publications related to factors influencing health insurance purchase intentions has steadily increased. This indicates a growing academic interest in this topic over the past decade.

## TCM framework-based review

**Fig. 3:**
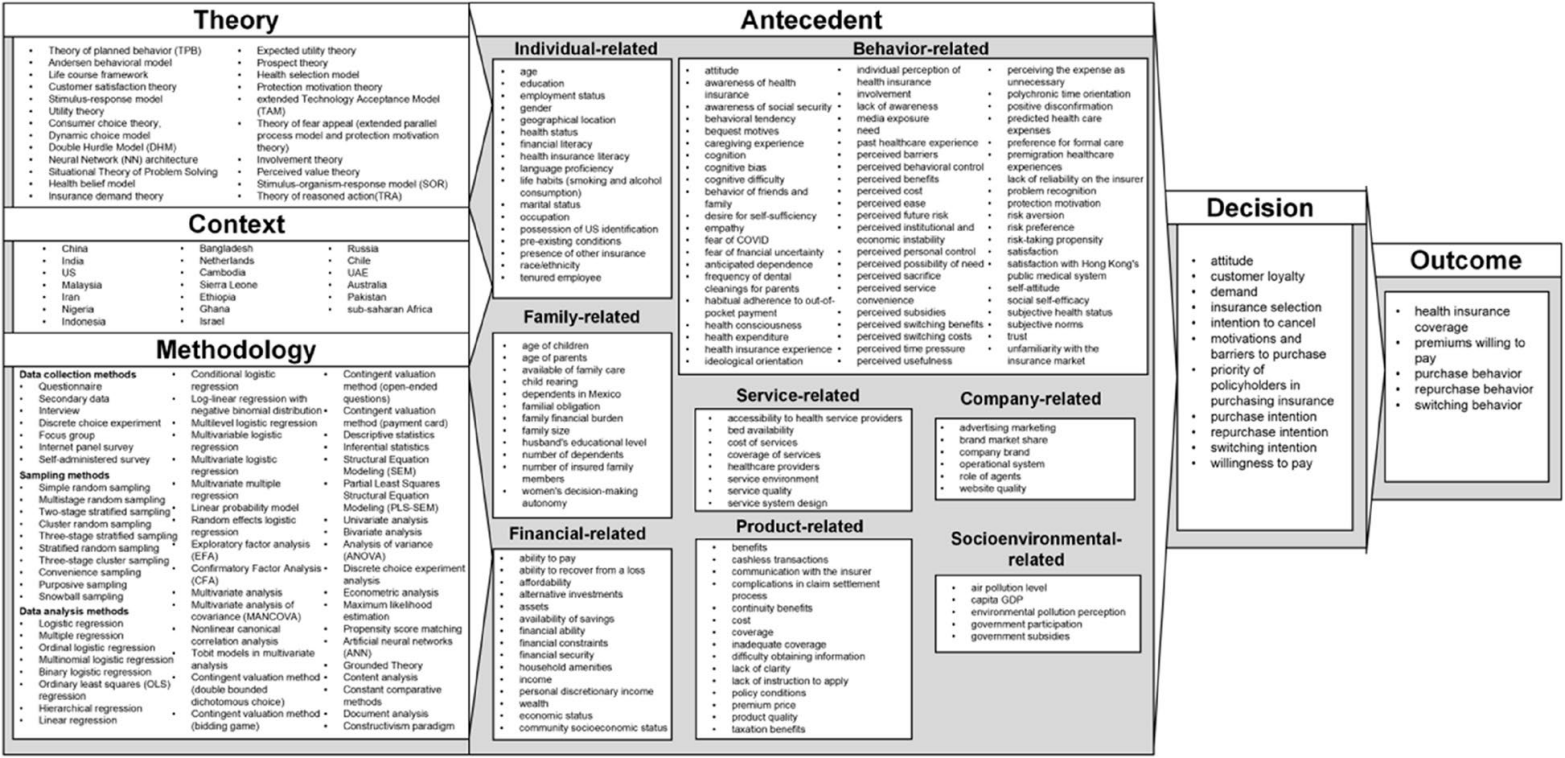
Overview of studies using TCM and ADO frameworks, adapted from Bhatia et al. (2021)

This section utilizes the TCM framework [43] to analyze the reviewed articles, summarizing key information on the theories, contexts, and methodologies used. Detailed insights are provided in Table 3 and Fig. 3.

**Table 3:** Overview of the 48 reviewed articles

Article	Theory	Context		Method		
		Country	Insurance type	Data collection	Sampling	Data analysis
Babu and Kaur [24]	N/A	India	Health insurance	Questionnaire; Secondary data	Convenience sampling	Descriptive statistics; Exploratory factor analysis; Inferential statistics
Block et al. [30]	N/A	US	bi-national health insurance	Questionnaire	N/A	Logistic regression
Gupta and Trivedi [46]	N/A	India	Health insurance	Questionnaire	Convenience sampling	Contingent valuation method; Ordinary least squares regression
Nosratnejad et al. [47]	N/A	Iran	Social health insurance	Questionnaire	Random sampling	Contingent valuation method
Wen et al. [48]	N/A	China	Oral health insurance	Questionnaire	Convenience sampling	Multiple regression; Inferential statistics
Ahmed et al. [49]	N/A	Bangladesh	Community-based health insurance	Questionnaire	Random sampling	Contingent valuation method; Multiple regression
Allaire et al. [27]	N/A	US	Long-term care insurance	Discrete choice experiment; Internet panel survey	Convenience sampling	Standard discrete choice econometric techniques; Descriptive analysis
Broyles et al. [50]	Life course framework	US	Long-term care insurance	Focus group	Convenience sampling	Content analysis
Duijmelinck and Van De Ven [51]	N/A	Netherlands	Health insurance	Questionnaire; Secondary data; Internet panel survey	Convenience sampling	Bivariate analysis; Binary logistic regression
Fitzgerald and Bias [52]	Customer satisfaction theory	US	Health insurance	Questionnaire	Convenience sampling	PLS-SEM
Kamimura et al. [53]	N/A	US	Health insurance	Self-administered survey	Convenience sampling	Descriptive statistics; Inferential statistics; ANOVA; MANCOVA; Multivariate multiple regression
Lin and Prince [54]	N/A	US	Long-term care insurance	Secondary data	N/A	Linear probability model
Mathur et al. [55]	N/A	India	Health insurance	Questionnaire	Convenience sampling	SEM
Nosratnejad et al. [56]	N/A	Iran	Health insurance	Secondary data	N/A	Ordered logistic regression
Ozawa et al. [31]	N/A	Cambodia	Community-based health insurance	Discrete choice experiment	Cluster random sampling	Discrete choice experiment analysis; Conditional logistic regression; Inferential statistics
Reid et al. [57]	N/A	US	Health insurance	Secondary data	N/A	Conditional logistic regression
Adedeji et al. [58]	N/A	Nigeria	Community-based health insurance	Questionnaire	Simple random sampling; Cluster random sampling	Descriptive statistics; Inferential statistics
He [59]	N/A	China (HK)	Health insurance	Questionnaire	Random sampling	Univariate and multivariate analysis; Logistic regression
Jayaraman et al. [60]	Stimulus-response model	Malaysia	Health insurance	Questionnaire	N/A	Descriptive statistics; Confirmatory factor analysis; Multiple regression analysis; Nonlinear canonical correlation analysis
Brahmana et al. [29]	Theory of planned behavior	Indonesia	Health insurance	Questionnaire	Convenience sampling	PLS-SEM
Jofre-Bonet and Kamara [61]	N/A	Sierra Leone	Health insurance	Questionnaire	3-stage stratified sampling	Contingent valuation method; Descriptive statistics; Univariate and bivariate descriptive analysis
Kassahun et al. [62]	Utility theory	Ethiopia	Village-based health insurance	Questionnaire	Multistage random sampling	Bivariable and Multivariable logistic regression
Khalid and Serieux [63]	Consumer choice theory	Ghana	Health insurance	Secondary data	N/A	Propensity score matching; Descriptive statistics
Mathur et al. [64]	N/A	India	Health insurance	Questionnaire	Multistage random sampling	SEM; Exploratory factor analysis; Linear regression; Descriptive statistics; Multivariate analysis
Wang et al. [21]	N/A	China	Long-term care insurance	Questionnaire	2-stage stratified sampling	Contingent valuation method; Document analysis; Random effects logistic regression
Hagani et al. [65]	N/A	Israel	Supplementary health insurance	Focus group; Questionnaire	Random sampling	Log-linear regression; Standard univariate analysis
Kaneva et al. [28]	N/A	Russia	Cooperative health insurance	Questionnaire	Multistage random sampling	Contingent valuation method; Ordinary least squares; Ordered logit; multinomial logit regression
Ogundeji et al. [25]	N/A	Nigeria	Social health insurance	Questionnaire	3-stage cluster sampling	Contingent valuation method; Logistic regression
Pardo [66]	Dynamic choice model	Chile	Health insurance	Secondary data	N/A	Ordinal logistic regression; Maximum likelihood estimation
Tolani et al. [67]	Double Hurdle Model; Neural Network architecture	UAE	Social health insurance	Secondary data	N/A	Artificial neural networks (ANN); Linear regression
Xu et al. [68]	Andersen behavioral model	China	Long-term care insurance	Questionnaire	Convenience sampling	Descriptive statistics; Bivariate analysis and multivariate logistic regression
Abdel Fattah et al. [69]	N/A	Malaysia	Health insurance	Questionnaire	Convenience sampling	PLS-SEM
Mamun et al. [70]	Theory of planned behavior	Malaysia	Health insurance	Questionnaire	Convenience sampling	PLS-SEM
Tam et al. [71]	Situational Theory of Problem Solving	Australia	Health insurance	Questionnaire	Convenience sampling	Exploratory Factor Analysis; Confirmatory Factor Analysis; SEM
Ullah et al. [72]	N/A	Pakistan	Maternal health insurance	Questionnaire	Random sampling	Logit regression; Logistic regression
Uma and Ilango [73]	Health belief model	India	Health insurance	Questionnaire	Convenience sampling	Inferential statistics; Logistic regression
Wang et al. [74]	Insurance demand theory	China	Health insurance	Secondary data	N/A	Multinomial logistic regression
Yeh et al. [75]	Expected utility theory; Prospect theory	China (TW)	Long-term care insurance	Questionnaire	Convenience sampling	Multinomial logistic regression; Descriptive analysis; Bivariate analysis
Jeong et al. [76]	Health selection model	US	Health insurance	Interview	Convenience sampling	Constant comparative methods rooted in Grounded Theory
Misra et al. [77]	Protection motivation theory	India	Health insurance	Questionnaire	Snowball sampling	PLS-SEM
Sunjaya et al. [32]	N/A	Indonesia	Health insurance	Interview	Random sampling	Grounded Theory; Constructivism paradigm
He et al. [26]	Andersen behavioral model	China (HK)	Long-term care insurance	Discrete choice experiment	2-stage stratified sampling	Descriptive statistics; Tobit models in multivariate analysis
Jadhav and Ramakrishna [78]	Theory of planned behavior	India	Health insurance	Interview	Convenience sampling	Content analysis
Lee [33]	extended Technology Acceptance Model	China (TW)	Health insurance	Questionnaire	Purposive sampling	ANOVA; Multiple regression; Hierarchical regression
Saraf and Baser [79]	Theory of fear appeal	India	Health insurance	Questionnaire	N/A	Econometric analysis; Binary logistic regression
Shen et al. [80]	Involvement theory; Perceived value theory	China	Health insurance	Questionnaire	Stratified random sampling	Exploratory factor analysis; ANOVA; Standard multiple regression; Hierarchical multiple regression
Sun et al. [81]	Stimulus-organism-response model; Theory of reasoned action	China	Health insurance	Questionnaire	2-stage stratifed sampling	SEM; Exploratory factor analysis; Confirmatory factor analysis; Descriptive statistics
Zegeye et al. [23]	N/A	sub-saharan Africa	Health insurance	Secondary data	N/A	Descriptive analysis; Inferential statistics; Bivariate and multilevel logistic regression

Among the 48 articles reviewed, 26 did not specify any theoretical framework, while the remaining 22 employed 23 different theories (Table 3). The most frequently used theory was the Theory of Planned Behavior (TPB), appearing in three articles, followed by the Andersen Behavioral Model in two articles. Each of the other theories was featured in only one study, reflecting a highly fragmented theoretical landscape. These theories span multiple disciplines, including health, economics, consumer behavior, technology adoption, and psychological responses to stimuli and life events. The frequent use of TPB highlights its relevance in explaining health insurance decisions, particularly in contexts involving financial planning or risk aversion where behavioral intentions are critical. However, its limited overall usage suggests no single theory dominates the field, underscoring the multifaceted nature of health insurance behavior and the need for interdisciplinary approaches. The absence of a theoretical framework in over half of the studies signals a significant gap, emphasizing empirical observation over theoretical grounding, which may limit generalizability and the depth of insights.

This study examines countries and insurance types as key research contexts (Table 3), reflecting societal, economic, and political dynamics [44]. China is the most frequently studied country, with 10 articles (6 from Mainland China, 2 from Hong Kong, and 2 from Taiwan), likely due to its evolving healthcare system and aging population. The United States and India each feature in 8 studies, reflecting their large populations and diverse healthcare markets. Malaysia, Iran, Nigeria, and Indonesia (2–3 articles each) represent a growing interest in emerging markets with expanding insurance sectors. The focus on large-population countries and growing insurance markets reflects research attention on health insurance adoption in rapidly evolving economic and policy contexts. This distribution highlights the need to examine smaller or low-income nations to broaden cross-country comparisons and enhance the understanding of global trends.

The review also identifies ten health insurance subtypes. General private health insurance dominates (29 articles), highlighting its relevance in both developed and emerging markets. Long-term care insurance (7 articles) reflects rising concerns about aging, while social health insurance and community-based health insurance (3 articles each) address regional public health needs. The prevalence of private health insurance studies underscores global interest in individual market-driven decisions, whereas niche types, such as long-term care insurance, signal a growing focus on aging populations and vulnerable groups. Future research could delve deeper into these subtypes to inform targeted policy interventions and market innovations.

Methodological analysis of the 48 studies reveals key trends and limitations (Table 3). Most studies relied on primary data (43 articles), with questionnaires being the dominant tool (32 articles), reflecting an emphasis on direct respondent engagement but also posing risks of response bias and limited contextual depth. The preference for questionnaires likely stems from their efficiency and suitability for large samples, but richer insights could be gained through interviews or focus groups. Non-probability sampling, particularly convenience sampling (18 articles), was common, potentially compromising generalizability. The frequent use of convenience sampling may be due to resource constraints or participant accessibility challenges, but adopting more robust probability sampling methods, such as simple random sampling, could improve external validity, particularly in diverse or emerging markets.

Quantitative analysis, particularly regression techniques (35 articles), dominates, reflecting a preference for predictive modeling. However, the minimal use of advanced techniques such as artificial neural networks (ANN) indicates an opportunity to explore more complex relationships. Qualitative analysis is underrepresented (7 articles), with methods like grounded theory rarely used, limiting behavioral insights. The reliance on regression-based approaches likely reflects their familiarity and reliability for testing variable relationships, but incorporating advanced models and qualitative methods could enrich analyses by capturing nonlinear patterns and in-depth decision-making processes. A mixed-methods approach could bridge this gap, offering both predictive insights and richer contextual understanding.

## ADO framework-based review

This section presents the antecedents (drivers or barriers), decisions, and outcomes using the ADO framework, [82] as visually depicted in Fig. 3. A voting methodology [83] is also applied to quantify the relationships between antecedents influencing health insurance purchase intentions, and their corresponding decisions and outcomes, as summarized in Table 4.

**Table 4:** Relationship map of the ADO in purchase intention toward health insurance context (Kahiya, 2018)

	Decision																
	Attitude			Customer loyalty			Demand			Insurance selection			Intention to cancel			Motivations and barriers to purchase	
Antecedent	+	0	-	+	0	-	+	0	-	+	0	-	+	0	-	+	0
Individual-related	0	0	0	0	0	0	1	2	1	3	0	1	1	0	0	0	0
Family-related	0	0	0	0	0	0	0	0	0	0	0	0	0	0	0	0	0
Financial-related	0	0	0	0	0	0	1	0	0	0	0	0	0	0	0	1	0
Behavior-related	8	1	0	2	0	0	0	0	0	0	1	0	0	0	6	0	0
Product-related	0	0	0	0	0	0	0	0	1	0	0	0	0	0	0	4	0
Service-related	0	0	0	1	0	0	0	0	0	0	0	0	0	0	0	0	0
Company-related	0	0	0	0	0	0	0	0	0	0	0	0	0	0	0	0	0
Socioenvironmental-related	0	0	0	0	0	0	0	0	0	0	0	0	0	0	0	0	0
Total	8	1	0	3	0	0	2	2	2	3	1	1	1	0	6	5	0

	Decision																Outcome
	Motivations and barriers to purchase	Priority of policyholders in purchasing insurance			Purchase intention			Repurchase intention			Switching intention			Willingness to pay			Health insurance coverage
Antecedent	-	+	0	-	+	0	-	+	0	-	+	0	-	+	0	-	+
Individual-related	0	3	0	1	45	19	8	1	3	0	3	0	1	26	13	2	3
Family-related	0	0	0	0	5	2	7	0	0	0	0	0	0	2	1	1	2
Financial-related	1	1	0	0	14	1	3	0	0	0	0	0	0	9	0	1	3
Behavior-related	2	0	0	0	60	8	11	9	2	1	2	0	3	5	0	2	3
Product-related	2	0	0	0	7	0	8	2	0	0	0	0	0	0	0	1	0
Service-related	0	1	0	0	6	1	1	3	0	0	0	0	0	0	0	0	1
Company-related	0	0	0	0	4	0	0	2	0	0	0	0	0	0	0	0	0
Socioenvironmental-related	0	2	0	0	3	2	0	0	0	0	0	0	0	0	0	0	0
Total	5	7	0	1	144	33	38	17	5	1	5	0	4	42	14	7	12

	Outcome														Total		
	Health insurance coverage		Premiums willing to pay			Purchase behavior			Repurchase behavior			Switching behavior			Positive	Insignificant	Negative
Antecedent	0	-	+	0	-	+	0	-	+	0	-	+	0	-	+	0	-
Individual-related	3	0	34	15	3	52	19	10	1	3	0	3	0	1	176	77	28
Family-related	1	0	2	1	1	5	2	7	0	0	0	0	0	0	16	7	16
Financial-related	0	0	11	0	2	15	1	3	0	0	0	0	0	0	55	2	10
Behavior-related	0	0	7	0	4	68	10	17	9	2	1	2	0	3	175	24	50
Product-related	0	0	0	0	2	7	0	8	2	0	0	0	0	0	22	0	22
Service-related	0	0	0	0	0	7	1	1	3	0	0	0	0	0	22	2	2
Company-related	0	0	0	0	0	4	0	0	2	0	0	0	0	0	12	0	0
Socioenvironmental-related	0	0	0	0	0	5	2	0	0	0	0	0	0	0	10	4	0
Total	4	0	54	16	12	163	35	46	17	5	1	5	0	4	488	116	128

### Antecedent

In this study, 141 antecedents influencing the decisions and outcomes associated with the intention to purchase health insurance were identified from a review of 48 articles. To facilitate discussion, these antecedents are categorized into eight groups: *individual-*,* family-*,* financial-*,* behavior-*,* product-*,* service-*,* company-*, and *socioenvironmental-related antecedents*. The categorization is based on the intrinsic characteristics of each factor and its role in shaping decision-making. For example, individual-related factors capture personal attributes like age and health literacy, while financial-related factors emphasize affordability and income. This structured approach offers a comprehensive analysis of the diverse influences on health insurance adoption, drawing from the categorization method of Bhatia et al. [10]. Each category is subsequently discussed in detail.

#### Individual-related antecedents

Table 4 presents 204 associations among individual-related antecedents. Age shows varied effects on insurance intentions, with 19 associations indicating both positive [23, 53, 63, 65] and negative impacts [21, 30, 68, 72, 73]. This suggests that its influence changes with age and is further shaped by regional healthcare systems and economic stability. For example, in high-income countries, younger individuals may delay insurance purchase due to robust public health systems, while in developing regions, older populations may seek coverage as a necessity due to limited public support. Research comparing distinct age groups reveals significant differences, though the effect is non-linear [57, 59].

Education plays a crucial role, with 16 studies showing positive effects [23, 24, 56, 62] and 2 studies indicating negative effects [25, 26]. Higher education levels generally increase the likelihood of possessing health insurance, though some highly educated individuals may perceive less need for it, believing they can better manage risks independently. This reflects cultural and socio-economic attitudes towards self-sufficiency and institutional trust.

Gender has a non-linear impact, with 11 associations mainly examining gender-based differences in purchase intentions [26, 27, 60, 63, 71]. In regions with higher gender disparities, women’s autonomy in financial decisions can affect insurance adoption, as observed in studies from sub-Saharan Africa and South Asia [23].

Geographic location affects purchase intentions in 7 studies, revealing diverse outcomes based on factors such as urban versus rural residency and regional variations [25, 49, 57, 61, 81]. This highlights the importance of location-specific healthcare access and socio-economic disparities, as rural areas often face limited healthcare infrastructure.

Marital status, discussed in 7 articles, impacts purchase intentions non-linearly, reflecting changes in considerations post-family formation [26, 28, 61]. The effect may also differ culturally, with collectivist societies placing more emphasis on family well-being, thus encouraging joint family insurance plans.

Presence of other insurance, covered in 6 studies, shows both positive [48, 51, 59] and negative effects [26, 30, 66]. Positive impacts raise awareness and willingness to buy, while negative impacts suggest these policies might act as substitutes, reducing the perceived need for health insurance. This variation could stem from differences in public versus private insurance schemes across countries.

Employment status, explored in 5 articles, influences willingness to purchase health insurance, with positive effects observed in various contexts [47, 63]. Employment is linked to increased insurance acquisition, particularly among women and government sector employees [23, 30, 56]. In regions with employer-sponsored insurance systems, employment often determines access to affordable coverage.

Knowledge and literacy show mixed results, with financial literacy positively impacting purchase intentions in 2 studies [54, 74] and health insurance literacy positively influencing intentions in 3 studies [58, 64, 70]. These findings highlight the need for culturally tailored educational programs, as financial literacy levels vary widely across regions.

Health status is positively associated with purchase intentions in 4 studies [28, 61, 66, 67]. Individuals with better health are more likely to purchase insurance, while those with pre-existing conditions often face difficulties finding suitable coverage. In some contexts, such as the United States, pre-existing conditions can be a major barrier due to policy restrictions, whereas universal healthcare regions show fewer such disparities.

Occupation shows varying willingness across different job types, as discussed in 4 articles [25, 49, 61, 74]. Jobs in the public sector are more likely to offer employer-sponsored health plans, reflecting economic and policy differences.

Race/ethnicity has a notable impact, highlighted in 2 studies [57, 65]. In multicultural contexts, historical inequities in access to healthcare services can influence minority groups’ likelihood of purchasing insurance. Other individual-related antecedents are mentioned in only one study each.

#### Family-related antecedents

Family-related antecedents total 12 variables, with Table 4 showing 32 associations. Four studies emphasize family size’s impact on health insurance purchase intentions: one study found a negative effect due to higher premiums for larger families, [47] while other studies observed a positive effect due to increased concern for family health [25, 31, 62]. In regions where family units are larger due to cultural norms, concerns about financial protection for dependents can strongly drive demand.

Regarding family care availability, three studies found it negatively impacts purchase intentions [24, 26, 68]. Additionally, factors like child-rearing responsibilities [50] and family obligations [26] also negatively affect willingness, likely due to perceived family care substituting formal insurance needs. This is more prominent in collectivist cultures where extended family support systems reduce the perceived need for insurance coverage. Other variables are addressed in individual studies.

#### Financial-related antecedents

Financial-related antecedents include 15 variables focused mainly on income and wealth, with Table 4 detailing 65 associations. Of the 16 studies examining income’s impact on health insurance purchase intentions, 15 found a positive correlation, indicating that higher income generally increases willingness to buy [30, 49, 74, 79]. This reflects socio-economic inequalities, as lower-income populations in developing regions often face affordability barriers despite strong intentions to purchase insurance. A positive effect of personal discretionary income was also noted in a previous study, [75] while another study [72] observed a negative correlation among pregnant women, suggesting that high-income individuals may not perceive insurance as necessary for childbirth costs.

Wealth positively influences purchase intentions in four studies, [23, 56, 62, 63] highlighting that wealthier households are more likely to seek financial protection. This reinforces the importance of addressing income inequality in policy design to ensure broader coverage. Other variables are addressed in single studies.

#### Behavior-related antecedents

Behavior-related antecedents, encompassing 63 variables such as attitude, motivation, cognition, perception, risk, and social influence, collectively impact health insurance purchase intentions, with Table 4 summarizing 225 associations. These antecedents are influenced by cultural beliefs, policy environments, and individual life experiences.

Perception includes 17 antecedents with both positive and negative impacts on purchase intention. Positive factors include perceived behavioral control, [29, 70] perceived ease, [33] perceived future risk, [29] perceived institutional and economic instability, [50] perceived benefits, [69, 71, 73, 80] perceived switching benefits, [51] perceived possibility of need, [68] perceived service convenience, [55] perceived subsidies, [52] and perceived usefulness [29, 33, 70, 77]. Negative factors include perceived barriers and costs, [73] perceived switching costs, [51] perceived personal control, [67] perceived sacrifice, [80] perceived time pressure, [55] and perceptions of unnecessary expense [78]. These perceptions can vary by geography; for instance, financial instability may amplify perceived barriers in low-income countries, while in high-income regions, convenience-related concerns may dominate.

Attitude-related antecedents, covered in seven articles, show a positive correlation between attitude toward behavior and purchase intention [27, 29, 33, 58, 70, 81]. Self-attitude also positively influences purchase intention [32]. Cultural values around self-reliance versus reliance on social safety nets shape these attitudes. In collectivist cultures, positive attitudes toward social protection schemes can enhance uptake.

Experience-related antecedents are discussed in seven articles. Positive impacts of health expenditure [48, 79] and health insurance experience [55, 71] on purchase intention are noted. Past healthcare experience [46, 56] and premigration healthcare experiences [76] also positively influence intentions, especially among immigrant populations navigating new healthcare systems.

Awareness-related antecedents are examined in six articles. Awareness of health insurance and social security has a positive impact, [32, 54, 67, 72] while lack of awareness negatively impacts purchase intentions [78]. In regions with limited public outreach, gaps in awareness may perpetuate disparities in coverage.

Subjective norms are discussed in five studies, with four showing a positive impact on willingness to purchase health insurance [29, 33, 70, 73]. The positive influence of friends’ and family’s behavior is particularly notable in close-knit communities [81].

Trust-related antecedents, covered in five studies, show a positive impact on purchase intention [26, 64, 71, 73]. Lack of institutional reliability negatively affects purchase intentions, [24] highlighting the critical role of institutional trust in fostering consumer confidence.

Cognition-related antecedents are discussed in four articles. Positive impacts of cognition, [81] problem cognition, [71] and cognitive biases [28] are noted. Overestimating benefits increases insurance purchases, while underestimating reduces demand. Conversely, cognitive difficulty negatively impacts purchase intentions [26].

Subjective health status is addressed in two studies, with conflicting findings. One study suggests that worse subjective health leads to higher insurance intentions, indicating adverse selection, [59, 63] while another finds better self-reported health correlates with higher intentions, showing no adverse selection [51].

Satisfaction positively impacts purchase intentions [52, 55, 69]. It was found that satisfaction with the public medical system positively affects low-risk consumers’ insurance intentions and negatively affects high-risk consumers’ [59].

Anticipated dependence positively influences purchase intention, [26, 68] with a desire for self-sufficiency also having a positive impact. Individuals who prefer to be less dependent on others and anticipate higher dependency are more likely to purchase long-term care insurance.

Risk-related antecedents show mixed effects. Risk aversion positively impacts purchase intentions, [28] while risk preference also has a positive effect [27]. Conversely, risk-taking propensity negatively affects purchase intentions [75]. In contexts of financial uncertainty, even risk-tolerant individuals may opt for insurance as a precautionary measure.

Some variables appear in only two studies. Bequest motives, [26, 54] health consciousness, [29, 71] fear of COVID, [77] and fear of financial uncertainty [79] are positively linked to purchase intentions. These findings suggest that psychological and environmental stressors, such as health crises, can significantly shift consumer priorities. Consumer involvement is a significant predictor, with higher engagement correlating with greater intention [71, 80]. Other antecedents are mentioned in only one study each.

#### Product-related antecedents

Product-related antecedents include 15 variables, with Table 4 showing 44 associations. Three studies reported that the cost of insurance products negatively impacts purchase intentions, [53, 57, 64] with premium price similarly correlating negatively with purchase intentions in two studies [21, 31]. Coverage scope positively affects purchase intentions according to three studies, [31, 60, 78] although inadequate coverage was also found to have a negative impact [78]. This reflects regional differences in consumer expectations for coverage, as populations in low-income areas may prioritize affordability over comprehensive benefits.

Product quality is another key antecedent, positively affect purchase intentions in two studies [24, 57]. This factor is influenced by service availability and cultural expectations. Understanding differences in what consumers perceive as ‘quality’ can guide tailored policy offerings. Insurance benefits, including continuity and taxation benefits, also positively influenced purchase intentions [57, 64, 78]. Effective communication with insurers was found to enhance purchase willingness [31, 55]. Other variables were covered in individual studies.

#### Service-related antecedents

Service-related antecedents, totaling eight variables, are illustrated in Table 4 with 24 associations. Four studies identified that high service quality significantly boosts health insurance purchase intentions [30, 32, 64, 69]. The accessibility of healthcare providers and bed availability positively influence purchase intentions [24, 74]. Effective service system design and positive perceptions of healthcare providers and the service environment also enhance purchase willingness [55, 64]. Conversely, high costs associated with services negatively impact willingness to purchase health insurance, similar to the cost of insurance products [30].

These factors vary significantly across geographic regions, where rural areas may lack accessible providers, diminishing the perceived value of insurance. In some countries, provider reputation and ease of claim settlement are critical service dimensions, as seen in studies from Southeast Asia and sub-Saharan Africa [22, 23]. These differences suggest that improving service system design and reducing transactional complexities could lower barriers to access, enhance trust, and increase participation in health insurance programs.

#### Company-related antecedents

Six company-related antecedents, detailed in Table 4 with 12 positive associations, are each highlighted in a single study. Effective agent performance enhances health insurance purchase intentions, [24] while impactful advertising and marketing increase these intentions [81]. A larger brand market share positively influences consumer choices, [57] and a strong brand reputation boosts purchasing intentions [55]. The quality of a company’s website also positively affects consumer experience and purchase intentions, [52] supported by findings that operational systems positively influence these intentions [32].

These findings suggest that trust in companies and their communication channels is shaped by national regulations and cultural perceptions of corporate transparency. For example, in regions where insurance fraud is a concern, consumer trust in digital services may be lower, necessitating enhanced transparency measures such as detailed policy disclosures and user-friendly claim processes to built trust and improve adoption rates.

#### Socioenvironmental-related antecedents

Five socioenvironmental-related antecedents, detailed in Table 4 with 10 positive associations, involve variables related to government involvement and environmental perception. Air pollution significantly influences children’s health insurance purchase intentions, as greater pollution heightens concerns and willingness to buy [74]. Residents’ perception of environmental pollution also boosts purchase intentions [81]. Government involvement and subsidies positively impact purchasing intentions, with strong government support and subsidies encouraging insurance purchases [75, 80]. Additionally, higher capita GDP correlates positively with health insurance purchasing intentions [74].

These findings demonstrate how socio-political stability and economic conditions create significant variations in consumer health insurance behavior across countries. Government-sponsored education and outreach campaigns can address disparities in awareness, particularly in areas with limited insurance literacy. This underscores the importance of policies adapted to local socio-political contexts and highlights the need to integrate socioenvironmental factors into insurance strategies and future research to enhance the effectiveness of targeted interventions.

### Decision

It has been asserted that decisions are behavioral reactions to the process of making a purchase choice [82]. This study focuses on purchase intention toward health insurance as the primary decision variable, but also examines related variables such as attitudes, demand, insurance selection, intention to cancel, switching intention, repurchase intention, and willingness to pay. In total, 11 decision variables are considered, with Table 4 detailing the relationships between these variables and their antecedents.

Purchase intention is the most frequently used decision variable, with 182 references in Table 4. It is influenced by antecedents from all eight categories, with behavior-related antecedents being the most significant (71 references), followed by individual-related (53), financial-related (17), product-related (15), and family-related antecedents (12). Other categories received fewer than ten references each. This underscores that purchase intention serves as a core construct in health insurance research due to its predictive power across diverse antecedents. However, to capture more nuanced patterns, future studies could benefit from integrating qualitative insights and considering the impact of socio-cultural and economic contexts through mixed-method approaches.

Willingness to pay is the second most common decision variable, with 49 references, mainly linked to individual-related (28) and financial-related antecedents (10). This indicates its suitability for examining consumer intentions in health insurance research, especially with individual and financial antecedents. Willingness to pay is typically measured using contingent valuation methods in questionnaires, reflecting a strong focus on economic valuation. This highlights the importance of understanding consumer valuation, but also suggests that further research should incorporate variables such as perceived trust and service convenience, which may vary across demographic and cultural settings. Most other decision variables appeared only once or twice across studies, indicating that their influence remains underexplored in the literature.

### Outcome

Outcomes are the consequences of consumer behavioral reactions [82]. While many studies focus on decision variables, they often do not explore outcome variables in depth. This study identified five outcome variables. The most common are purchasing behavior and willingness to pay premiums, with 209 and 66 references, respectively (Table 4). This is followed by repurchase behavior (18 votes), health insurance coverage (12 votes), and switching behavior (9 votes).

The emphasis on initial engagement metrics, such as purchasing behavior and premiums, suggests a strong interest in consumer acquisition patterns. However, the limited focus on long-term metrics, such as retention and repurchase behavior, indicates the need for more longitudinal studies to assess sustained consumer engagement and switching behavior over time, particularly in dynamic markets influenced by policy and technological shifts. Incorporating such metrics could provide richer insights into the drivers of loyalty and the impacts of policy interventions on consumer retention and satisfaction.

## Directions for future research

### Future directions: TCM dimensions

Theoretically, future research should address the theoretical fragmentation in health insurance decision-making studies. First, further validation and extension of the TPB across diverse cultural and socioeconomic contexts is needed to assess its broader applicability. Additionally, integrating TPB with complementary models, such as risk perception or behavioral economics frameworks, could better capture the multidimensional factors influencing insurance decisions. Given the limited theoretical foundations in many studies, future research should develop and apply hybrid models that incorporate emerging factors like digital technologies and personalized insurance products. These efforts would enhance the generalizability and depth of findings, offering a more comprehensive understanding of health insurance behavior.

Contextually, studies should expand to include smaller or lower-income countries with underdeveloped or reforming health insurance systems to uncover unique challenges and opportunities in expanding coverage. Research should also focus on emerging markets like Malaysia, Iran, Nigeria, and Indonesia to examine how economic growth and regulatory changes influence insurance adoption. Comparative studies across these regions could identify common drivers and barriers. Additionally, with increasing attention on niche insurance types like long-term care and community-based health insurance, future research should explore their scalability and sustainability, particularly in meeting the needs of aging populations and underserved communities. Longitudinal studies could provide insights into their long-term effectiveness and adaptability.

Methodologically, future research should incorporate qualitative methods such as interviews and focus groups to complement the prevalent use of questionnaires. Mixed-methods approaches can provide richer insights into behavioral factors while enhancing the predictive value of quantitative models. Rigorous probability sampling techniques, such as random or stratified sampling, should be prioritized to improve the generalizability of findings, especially in diverse populations within emerging markets or lower-income countries. Moreover, as health insurance markets grow more complex, advanced data analysis techniques, like machine learning or ANN, should be explored to better model non-linear relationships and improve predictive accuracy. These steps would enhance methodological rigor and provide more comprehensive insights into health insurance behavior across diverse populations.

### Future directions: ADO dimensions

Given the substantial influence of demographics on insurance intentions, future research should examine how digital literacy and technology adoption within these groups impact health insurance decisions, aligning with the sector’s increasing digitization. Additionally, since health status is a critical factor, studies could explore personalized insurance products tailored to individual health needs to address the diverse challenges faced by people with varying health conditions. The impact of socioenvironmental factors like air pollution suggests the need to assess their broader effects on insurance policy design across different environmental contexts. The role of employment status highlights the importance of investigating how changing employment patterns, such as the gig economy, affect health insurance decisions. Similarly, family dynamics—including family size and caregiving responsibilities—warrant further research to inform the development of family-centric policies. Although current studies emphasize the importance of perceived future risks and instability, further exploration is needed to understand how these perceptions—and their underlying antecedents—affect consumers’ willingness to purchase health insurance.

Additionally, expanding research to include repurchase intention, switching intention, and recommendation intention could provide a more comprehensive understanding of post-purchase consumer behavior. Finally, although existing studies primarily focus on how antecedents influence decision-making, there is limited research on their direct and indirect effects on outcomes such as claim rates and long-term retention. Addressing these gaps in future studies could offer valuable insights for insurers and policymakers, despite the inherent challenges of data tracking and causal analysis.

### Future directions: summary

To clearly outline future research directions, Table 5 presents the research questions across the six dimensions discussed above. 

**Table 5:** Summary of suggested directions for future research

Dimension		Direction	Suggested research quesions
Theory	1	Validation of TPB in diverse contexts	How does TPB explain health insurance purchase behavior in different cultural and socioeconomic contexts?
	2	Integration of TPB with other models	Does integrating TPB with risk perception or behavioral economics frameworks improve predictions of health insurance decisions?
	3	Development of new or hybrid models	How can new or hybrid models capture the influence of digital technologies and personalized insurance products on health insurance choices?
Context	4	Lower-income countries	What are the key factors affecting health insurance expansion in underdeveloped systems, and how do they differ across regions?
	5	Emerging markets	How do economic growth and regulatory changes influence health insurance adoption in emerging markets, and what are the regional similarities?
	6	Niche insurance types	How scalable and sustainable are niche insurance models in meeting the needs of aging populations and underserved communities, and what are their long-term impacts?
Method	7	Qualitative methods	How can interviews and focus groups offer deeper insights into health insurance decision-making?
	8	Sampling techniques	How does probability sampling enhance the generalizability of health insurance behavior research in diverse markets?
	9	Advanced data analysis	How can machine learning and ANN improve the accuracy of predictive models for health insurance decisions?
Antecedent	10	Demographic variability and technology use	How do digital literacy and technology adoption across demographic groups affect health insurance purchase intentions?
	11	Health status and personalized insurance	What factors do individuals with different health conditions consider when choosing personalized health insurance?
	12	Socioenvironmental factors and policy implications	How do socioenvironmental factors like air pollution influence health insurance policy design and effectiveness?
	13	Impact of employment shifts	How do changing employment patterns, such as the gig economy, affect health insurance purchase decisions?
	14	Family dynamics and insurance decisions	How do family size and responsibilities influence health insurance purchasing decisions?
	15	Perceived future factors	How do perceived future risks impact consumers’ willingness to purchase health insurance?
Decision	16	Post-purchase recommendation intentions	What factors influence a consumer’s intention to recommend health insurance after purchase?
	17	Role of recommendation behavior	Does recommendation behavior significantly influence health insurance purchase decisions?
Outcome	18	Impact of purchase antecedents on insurers	How do health insurance purchase antecedents, like perceived risk and demographics, affect insurers’ claim rates and retention?

## Conclusion

This study provides a comprehensive review of the factors influencing health insurance purchase intentions using both the TCM and ADO frameworks. The TCM framework highlights significant theoretical fragmentation, with the TPB being the most frequently used model, but with many studies lacking a consistent theoretical basis. This underscores the need for integrating more robust and complementary models, such as those from behavioral economics and risk perception, to better capture the complexities of health insurance behavior [44, 82]. By combining TCM’s contextual analysis with ADO’s structured approach, this review presents an integrated perspective on how antecedents, decisions, and outcomes interact, providing insights that go beyond the narrow scope of single-framework studies.

Geographically, research remains concentrated in major economies like China, the United States, and India, while smaller or lower-income countries receive limited attention. This highlights a gap in understanding insurance behavior across diverse regions, reinforcing the need for cross-country comparative studies to explore unique challenges in health insurance adoption [38]. Additionally, niche insurance types remain underexplored, limiting insights into how specific populations, such as gig workers or aging adults, make purchase decisions. Recent studies highlight similar gaps in other areas, such as the adoption of natural products and healthcare apps, where demographic differences and socioenvironmental concerns play a crucial role [84, 85].

Methodologically, most studies rely on quantitative approaches, particularly regression analysis, with an underuse of qualitative methods and advanced techniques such as ANN. This points to the need for more diverse research designs that incorporate mixed-methods approaches and machine learning tools to capture complex consumer behaviors [79, 81]. Unlike previous reviews, this study emphasizes the importance of context-sensitive methodologies that account for cultural norms and socio-political conditions, enriching the understanding of purchase intentions.

Through the ADO framework, behavioral factors—such as attitudes, perceptions, and social influences—are identified as the most influential in shaping purchase intentions, followed by financial factors like income and wealth, demonstrating the strong role of economic conditions in decision-making. Individual factors like age and education show mixed effects across different demographics, while family dynamics (e.g., family size and caregiving responsibilities) and socioenvironmental factors (e.g., air pollution and government support) also play important roles in shaping insurance demand. This review extends prior research by synthesizing findings across contexts to highlight the interconnectedness of social, economic, and behavioral factors [10].

This review also addresses the fragmented use of models in health insurance research, emphasizing the need for integrating and validating complementary models that consider emerging factors, such as digital health technologies and personalized insurance products, to better capture the multidimensional nature of health insurance behavior [15, 41]. For example, studies on mobile health app adoption reveal that emotional value and conditional use significantly affect user engagement, [86] suggesting that insurance research should similarly incorporate digital convenience and perceived utility. Unlike studies that solely highlight limitations, this review provides specific guidance on hybrid models that combine behavioral and technological elements to improve predictive accuracy.

From a practical standpoint, this study offers actionable insights for policymakers and insurers. Expanding health insurance coverage in underserved regions requires tailored products that meet the needs of diverse demographic groups, including aging populations and low-income families. Targeted initiatives, such as mobile health platforms and community-based outreach, can improve accessibility and engagement [77]. The discussion also highlights the importance of addressing post-purchase behaviors, such as repurchase intention and switching behavior, by improving service delivery and fostering trust through personalized customer support. Efforts to improve health insurance literacy and reduce perceived risk are also crucial, as they can enhance brand reputation and strengthen purchase intentions [87].

By synthesizing key insights and positioning them within established frameworks, this review contributes to both academic research and practical policymaking. It provides a roadmap for enhancing health insurance adoption and retention, addressing disparities, and developing sustainable insurance systems, while offering a foundation for future research to build more context-specific, data-driven solutions.

However, this review has some limitations that should be acknowledged. Due to the non-availability of certain studies, some geographical contexts may be underrepresented, potentially influencing the generalizability of the findings. Additionally, while this review follows a structured synthesis approach, it does not include a formal quality assessment of the included studies, which may limit the robustness of methodological evaluations. Future research should incorporate systematic quality assessments and include a broader range of studies to provide more nuanced insights across diverse regions and insurance types.

## Data Availability

No datasets were generated or analysed during the current study.
